# Inhibition of FAK kinase activity preferentially targets cancer stem cells

**DOI:** 10.18632/oncotarget.18517

**Published:** 2017-06-16

**Authors:** Vihren N. Kolev, Winnie F. Tam, Quentin G. Wright, Sean P. McDermott, Christian M. Vidal, Irina M. Shapiro, Qunli Xu, Max S. Wicha, Jonathan A. Pachter, David T. Weaver

**Affiliations:** ^1^ Verastem, Inc., Needham, MA, USA; ^2^ Comprehensive Cancer Center, Department of Internal Medicine, University of Michigan, Ann Arbor, MI, USA

**Keywords:** cancer stem cells, FAK, focal adhesion kinase, VS-4718, VS-6063

## Abstract

Because cancer stem cells (CSCs) have been implicated in chemo-resistance, metastasis and tumor recurrence, therapeutic targeting of CSCs holds promise to address these clinical challenges to cancer treatment. VS-4718 and VS-6063 are potent inhibitors of focal adhesion kinase (FAK), a non-receptor tyrosine kinase that mediates cell signals transmitted by integrins and growth factor receptors. We report here that inhibition of FAK kinase activity by VS-4718 or VS-6063 preferentially targets CSCs, as demonstrated by a panel of orthogonal CSC assays in cell line models and surgically resected primary breast tumor specimens cultured *ex vivo*. Oral administration of VS-4718 or VS-6063 to mice bearing xenograft models of triple-negative breast cancer (TNBC) significantly reduced the proportion of CSCs in the tumors, as evidenced by a reduced tumor-initiating capability upon re-implantation in limiting dilutions of cells prepared from these tumors. In contrast, the cytotoxic chemotherapeutic agents, paclitaxel and carboplatin, enriched for CSCs, consistent with previous reports that these cytotoxic agents preferentially target non-CSCs. Importantly, VS-4718 and VS-6063 attenuated the chemotherapy-induced enrichment of CSCs *in vitro* and delayed tumor regrowth following cessation of chemotherapy. An intriguing crosstalk between FAK and the Wnt/β-catenin pathway was revealed wherein FAK inhibition blocks β-catenin activation by reducing tyrosine 654 phosphorylation of β-catenin. Furthermore, a constitutively active mutant form of β-catenin reversed the preferential targeting of CSCs by FAK inhibition, suggesting that this targeting is mediated, at least in part, through attenuating β-catenin activation. The preferential targeting of cancer stem cells by FAK inhibitors provides a rationale for the clinical development of FAK inhibitors aimed to increase durable responses for cancer patients.

## INTRODUCTION

Research over the last decade has led to the hypothesis that individual tumors contain diverse populations of neoplastic cells, including a minor subpopulation of cancer stem cells (CSCs) or tumor-initiating cells (TIC), having the capability for tumor-initiation, self-renewal, and potential to differentiate into the heterogeneous tumor cell populations present within tumors [1]. Additionally, CSCs are relatively resistant to standard chemotherapy, enabling them to survive initial therapy and spawn clinical relapses [2]. CSCs are also thought to exhibit an elevated ability to disseminate from primary tumors and generate metastases at distant anatomical sites [3]. For these reasons, the development of agents that effectively target CSCs appears to be essential for the clinical management of cancer and prevention of cancer recurrence.

Most current anticancer therapies target the bulk populations of cancer cells within tumors. Because they are less effective against CSCs, some CSCs may survive the initially employed therapeutic agents and generate tumor regrowth at primary and metastatic sites. Notably, in breast cancer patients following neo-adjuvant chemotherapy, the continued presence of CSCs in axillary lymph nodes identified by expression of the CSC marker aldehyde dehydrogenase-1 (ALDH1), was associated with poor overall survival (OS) [4]. In contrast, in patients with residual tumor in lymph nodes that lacked ALDH1-positive CSCs, OS was greatly improved and not significantly different from that in patients with no residual disease. Similar observations have been published by multiple groups and extended to other cancers including ovarian and non-small cell lung cancers [5–10]. Collectively, these studies provide clinical proof-of-concept for the importance of CSCs in patient prognosis, and suggest that combination of CSC-targeting agents with conventional bulk tumor cell-targeting therapy may be critical for meaningful improvement of long term survival of patients with cancer [11, 12]. Clinical trials with CSC-targeting agents have been prompted by these recent advances [13].

Focal adhesion kinase (FAK), a non-receptor tyrosine kinase, mediates transduction of signals released by integrins and growth factor receptors; once activated, it regulates diverse cellular functions, including adhesion, proliferation, migration and survival [14]. Multiple studies have demonstrated that FAK expression and activity are upregulated in many epithelial tumors and associated with poor patient prognosis [15–17]. Therefore, FAK has been pursued as a promising therapeutic target for cancer. Indeed, several small molecule FAK kinase inhibitors have been shown to decrease tumor growth and metastasis in preclinical models and advanced to clinical development [18, 19]. Recent studies have linked specific mutation dependency of cancer cells on integrin-FAK signaling, such as *NF2* tumor suppressor loss in mesothelioma and Ph+ B-cell acute lymphocytic leukemias that are especially sensitive to FAK inhibition [20, 21]. These studies provide biological rationale for FAK inhibitors in specific patient populations and cancer settings. Furthermore, FAK inhibition has also been shown to induce T cell-mediated tumor regression and enhance the efficacy of immune checkpoint inhibitors [22, 23].

The role of FAK in the self-renewal and tumor-initiating capabilities of cancer stem cells has been suggested by several reports. Loss of FAK in mouse epidermis resulted in suppression of progression of induced benign papillomas to malignant lesions [24]. Moreover, keratinocyte-specific FAK knockout resulted in occasional spontaneous regression of newly developing tumors, suggesting that either CSCs necessary for sustained tumor growth failed to generate, or the self-renewal of CSCs has been constrained [25]. Similarly, mammary cell-specific deletion of FAK suppresses tumorigenesis and depletes the CSC pool, as measured by stem cell markers, decreased tumorsphere formation, and impaired *in vivo* tumor initiation [26]. Both kinase-dependent and kinase-independent functions of FAK have been reported to be important for regulation of different pools of CSCs [27]. Although limited, these observations indicate the requirement of FAK kinase activity for CSC maintenance. However, the therapeutic utility of FAK kinase inhibitors as CSC-targeting agents has not been elucidated.

Given the crucial function of FAK signaling for self-renewal of CSCs, we hypothesized that inhibition of FAK kinase activity might suppress CSCs. VS-4718, previously known as PND-1186, is a potent and selective orally active FAK kinase inhibitor [28, 29]. VS-4718 inhibits tumor growth and metastasis in *in vivo* breast and mesothelioma cancer models [28, 29]. In the present study, we show that inhibition of FAK kinase activity preferentially targets breast CSCs *in vitro* and *in vivo*, while conventional chemotherapeutic agents, cisplatin and paclitaxel, increase the percentage of CSCs in cells surviving treatment. Importantly, VS-4718 prevented chemotherapy-induced enrichment of CSCs and tumor regrowth after cessation of chemotherapy in triple-negative breast cancer xenograft models. Mechanistic studies revealed that inhibition of FAK leads to inhibition of the Wnt/β-catenin pathway. Ectopic expression of non-degradable β-catenin blocked the reduction of CSCs by VS-4718, supporting the hypothesis that VS-4718 targets CSCs at least in part through blockade of the Wnt/β-catenin pathway. Our findings provide a rationale for combination of CSC-targeted agents with cytotoxic chemotherapy to effectively diminish both CSCs and non-CSC tumor cells. The development of these strategies may lead to more effective and less toxic therapies and, potentially, durable clinical responses.

## RESULTS

### Inhibition of FAK attenuates CSC function *in vitro*

**Figure 1:**
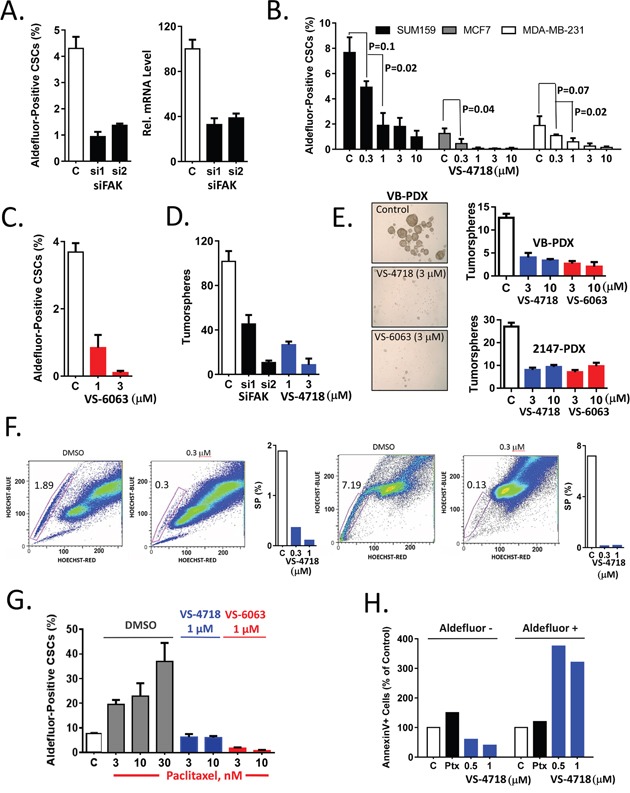
Inhibition of FAK signaling preferentially targets breast CSCs *in vitro* **(A)** siRNA-mediated knockdown of FAK resulted in decrease of the percentage of Aldefluor+ cells. MDA-MB-231 cells were transfected with 2 individual siRNAs against FAK. 48 h post transfection, cells were plated in matrigel and the percentage of Aldefluor+ cells was determined 2 days later. FAK knockdown was confirmed by RT-PCR, right panel. **(B)** VS-4718 dose-dependently reduced the percentage of Aldefluor+ tumor cells. SUM159, MCF7, MDA-MB-231 cell lines were treated with VS-4718 for 4 days in matrigel, and an imaging-based Aldefluor assay was carried out. **(C)** VS-6063 dose-dependently reduced the percentage of Aldefluor+ cells. MDA-MB-231 cells were treated with VS-6063 in matrigel and the percentage of Aldefluor+ cells determined. **(D)** siRNA-mediated ablation of FAK or FAK inhibition by VS-4718 reduced tumorsphere forming efficiency. MDA-MB-231 cells were transfected with FAK siRNA or treated with VS-4718 as in **(A)** or **(B)**. Cells were extracted from matrigel, plated for primary tumorspheres, and then re-plated for secondary tumorsphere assays. Number of secondary tumorspheres per 2000 cells are represented. **(E)** Cells from two TNBC PDX models –VB and 2147 were treated with VS-4718 or VS-6063 in matrigel and plated in primary and secondary tumorsphere assays as in **(D)**. **(F)** FAK inhibitor preferentially targeted SP CSCs. SUM159 and MCF7 cells were treated with VS-4718 in matrigel for 2 days. Cells were extracted from matrigel, incubated with Hoechst 33342 dye and SP was determined by FACS. Representative FACS data and quantitation are shown for SUM159 (left) and MCF7 (right). **(G)** VS-4718 and VS-6063 inhibit chemotherapy-induced Aldefluor+ CSCs. MDA-MB-231 were treated in matrigel with paclitaxel at indicated concentrations alone (DMSO) or in combination with either VS-4718 (1 μM) or VS-6063 (1 μM) for 4 days and the percentage of Aldefluor+ cells was determined. **(H)** VS-4718 induced apoptosis preferentially in CSCs. SUM159 cells were treated with paclitaxel (Ptx), VS-4718 or control for 24h, followed by co-staining with AnnexinV and Aldefluor reagents. Percentage of AnnexinV+ cells in Aldefluor- and Aldefluor+ populations was determined by FACS. Data presented in this figure are representative of 2 or 3 independent experiments.

We and others have demonstrated that subpopulations of breast cancer cells with higher ALDH1 enzymatic activity, as measured using an Aldefluor assay, are enriched for CSCs [6, 30]. To demonstrate the importance of FAK for CSC maintenance, we ablated FAK expression by FAK-specific siRNA introduction into MDA-MB-231 cells, and observed a significant diminution of the Aldefluor+ cells from 4.3% to ~1% of the tumor cell population (Figure 1A). We previously reported that inhibition of FAK activity has a strong effect on cell viability in 3D MoT (matrigel-on-top) culture [29]. Accordingly, we treated a panel of breast cancer cell lines growing in MoT culture with the VS-4718 FAK inhibitor and assessed its effects on CSCs using an Aldefluor assay. As indicated in Figure 1B, VS-4718 treatment resulted in dose-dependent reduction of the percentage of Aldefluor+ cells across three breast cancer cell lines. To further assess whether FAK kinase inhibition attenuates CSCs, we tested a second FAK kinase inhibitor, VS-6063, against CSCs. Similar to VS-4718, treatment of MDA-MB-231 cells with VS-6063 in MoT culture resulted in a dose-dependent decrease of the proportion of Aldefluor+ cells (Figure 1C). Thus, two structurally distinct FAK inhibitors, VS-4718 and VS-6063, preferentially target CSCs in breast cancer cell lines indicating that FAK kinase activity is important for the maintenance of CSCs.

We then employed a tumorsphere assay to determine whether FAK expression and kinase activity are necessary for CSC self-renewal and maintenance. MDA-MB-231 cells were either transfected with FAK siRNA and plated in MoT culture, or treated with VS-4718 in MoT culture for 4 days. Cells were extracted from matrigel and an equal number of cells were plated in drug-free medium in low adhesion plates to form tumorspheres. Since general cytotoxic drugs or cell cycle inhibitors may exert an effect on the primary tumorspheres, the primary tumorspheres were counted, dissociated and an equal numbers of live cells were then re-plated to assay for secondary tumorsphere formation. SiRNA-mediated knock-down of FAK resulted in more than 50% inhibition of primary tumorspheres (data not shown) and had an even stronger inhibitory effect (70%) on secondary tumorspheres, as did pharmacologic inhibition of the primary tumorsphere culture with VS-4718 (Figure 1D). Similarly, cells from two breast cancer patient-derived xenograft (PDX) models were treated with DMSO, VS-4718 or VS-6063 in MoT culture and then plated as tumorspheres. Pharmacological inhibition of FAK activity by VS-4718 and VS-6063 diminished the number of primary and secondary spheres in a dose-dependent manner (Figure 1E). These results indicate that FAK inhibitors suppressed CSCs during treatment in matrigel. The sustained inhibition of tumorsphere-forming ability further corroborates the Aldefluor+ cell inhibition observed, indicating that FAK kinase activity is important for CSC self-renewal.

CSC can also be identified by a Side Population (SP) assay that is defined by cellular exclusion of Hoechst 33342 dye. Several reports demonstrated that SP cells are enriched for CSCs in a wide range of cancers including breast cancer [30]. The SUM159 and MCF7 cell lines are derived from triple negative breast carcinoma and an ER+/PR+ tumor, respectively. We therefore sought to test the effect of a FAK inhibitor on SP cells by treating SUM159 and MCF7 human breast cancer cells in MoT culture for 4 days, staining SP cells with Hoechst 33342 dye followed by flow cytometric analysis. VS-4718 markedly reduced SP cells from 1.9% to 0.3% in SUM159 cells and from 7.2% to 0.1% in MCF7 cells (Figure 1F).

Collectively, these data from various CSC assays provide evidence that inhibition of FAK, achieved either by the VS-4718 or VS-6063 small molecule kinase inhibitors or by siRNA, preferentially targets CSCs in breast cancer cell lines, supporting the notion that FAK kinase activity is required for the maintenance of CSCs.

### VS-4718 attenuates chemotherapy-induced enrichment of CSCs

Chemotherapeutic agents have been reported to enrich for CSCs due in large part to the intrinsic resistance of CSCs to chemotherapy [31]. We have shown previously that the FAK inhibitor VS-4718 can abrogate the enrichment of mesothelioma CSCs resulting from exposure to the chemotherapeutic agent pemetrexed [29], but other forms of chemotherapy might differ with regard to CSC resistance. Thus, in this study we used paclitaxel, one of the standard chemotherapies used in breast cancer treatment. MDA-MB-231 breast cancer cells were treated with paclitaxel, alone or in combination with FAK inhibitors, and subjected to an Aldefluor assay to gauge the representation of CSCs in the treated cell populations. Paclitaxel caused a 2- to 4-fold increase in the proportion of Aldefluor+ cells. However, concurrent treatment with either VS-4718 or VS-6063 abolished this chemotherapy-induced increase of CSCs (Figure 1G), suggesting that FAK inhibitors may abrogate chemotherapy-induced enrichment of CSCs in breast cancer.

As a protective mechanism, breast CSCs may be more resistant to apoptosis, so the induction of apoptosis by FAK inhibitors was assessed in CSCs relative to non-CSCs. SUM159 human breast cancer cells were treated with VS-4718, paclitaxel, or with DMSO for 24h in MoT culture and the percentage of Annexin V-positive apoptotic cells was determined for both the Aldefluor+ and Aldefluor- subpopulations. VS-4718 induced more than 300% apoptosis in in Aldefuor+ cell population compared to control while no significant apoptosis was detected in Aldefluor- cell population (Figure 1H), indicating that VS-4718 preferentially induced apoptosis in CSCs. In contrast, paclitaxel did not show preferential induction of apoptosis in Aldefluor+ cells, although it did appear to induce 50% apoptosis in Aldefluor- non-CSCs and less than 10% apoptosis in Aldefluor+ cells. (Figure 1H). These data support the concept that effective targeting of relevant tumor cell subpopulations will require combination of chemotherapy with an anti-CSC agent, such as VS-4718.

### Inhibition of FAK activity attenuates CSCs in *ex vivo* primary breast cancer specimens and *in vivo* breast cancer animal models

**Figure 2:**
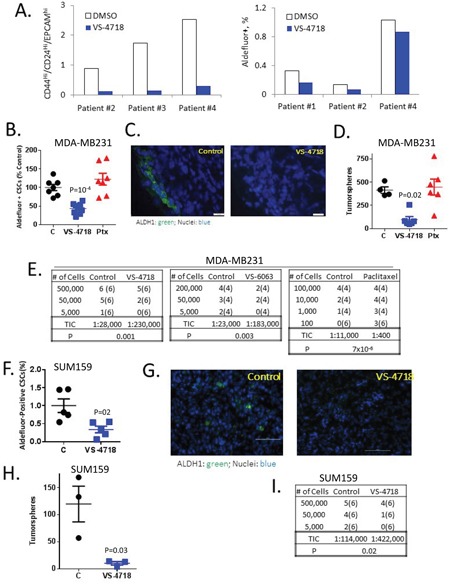
VS-4718 and VS-6063 preferentially abolish CSCs in *ex vivo* treated breast cancer samples and *in vivo* models **(A)** VS-4718 inhibits CSC populations in primary breast cancer specimens treated *ex vivo*. Breast tumor tissue was cut into small pieces, treated *ex vivo* with 1 μM VS-4718 for 5 days, and subjected to Aldefluor assay or EPCAM/CD24/CD44 FACS. **(B-I)** FAK inhibitors target CSCs *in vivo*. Mice bearing MDA-MB-231 tumors were treated with control, 100 mg/kg VS-4718 bid, 50 mg/kg VS-6063 bid, by oral gavage for 9 days or a single dose of 10 mg/kg paclitaxel (Ptx) by ip. Cells were dissociated from tumors and subjected to a panel of CSC assays. **(B, F)** VS-4718 preferentially reduced Aldefluor+ CSCs in MDA-MB-231 **(B)** and SUM159 **(F)** tumor xenografts. Shown is a scatter plot of % Aldefluor+ cells with each point representing one tumor. *P* = 0.002 (unpaired t-test). **(C, G)** Frozen sections from MDA-MB-231 **(C)** and SUM159 **(G)** tumors were stained for ALDH1 (green) and DAPI (blue). **(D, H)** VS-4718 but not paclitaxel (Ptx) reduced CSCs with self-renewal capability in MDA-MB-231 **(D)** and SUM159 **(H)** tumors. Dissociated cells were plated for tumorspheres and spheres were serially passaged for a third time (tertiary tumorspheres). Numbers of tertiary tumorspheres per 2000 cells are shown with each point representing one primary tumor. **(E, I)** *In vivo* limiting dilution assay results showed that VS-4718 and VS-6063 reduced the proportion of tumor-initiating cells (TIC) while paclitaxel enriched for TIC. Cells harvested from MDA-MB-231 **(E)** and SUM159 **(H)** tumors were xenografted in limiting dilutions into SHrN immunodeficient mice. Shown are the number of tumors formed. Number of mice implanted is shown in parenthesis. Tumor-initiating cells (TIC) were calculated using the ELDA software (http://bioinf.wehi.edu.au/software/elda/) based on the number of tumors formed by week 8.

Since our *in vitro* experiments suggested that inhibition of FAK can cause direct reduction of CSCs, we sought to demonstrate the effect of VS-4718 on CSCs in surgical specimens of primary patient tumors. CD44^hi^/CD24^lo^ and Aldefluor+ cells have been identified as breast CSCs and their presence predicts for poor survival [32]. Breast cancer tumor fragments from four patients were placed in MoT culture and treated with VS-4718 for five days. Tumor tissue was dissociated and the percentage of CD44^hi^/CD24^lo^ and Aldefluor+ cells was determined. VS-4718 treatment resulted in a substantial decrease of CD44^hi^/CD24^lo^ cells in the samples tested (Figure 2A, left panel), and some reduction in the percentage of Aldefluor+ cells (Figure 2A, right panel). Material from two patients was not sufficient to perform both CSC assays, so either CD44^hi^/CD24^lo^ or Aldefluor+ cells was determined. These data support the notion that CSC populations are evident in primary tumors from breast cancer patients, and further corroborate our *in vitro* findings that FAK inhibitors can reduce the proportion of CSCs.

To investigate the effect of FAK inhibitors or chemotherapy on CSCs *in vivo*, we treated mice bearing MDA-MB-231 (Figure 2B–2E) or SUM159 (Figure 2F–2I) triple-negative breast cancer (TNBC) tumors (200-400 mm^3^) with vehicle control (C), VS-4718, 100 mg/kg po bid for 10 days, VS-6063 50 mg/kg po bid for 10 days or paclitaxel 10 mg/kg ip at day 1. Under these conditions, the FAK inhibitors and paclitaxel did not lead to tumor volume reduction during the 10-day dosing period. Tumors then were dissociated to single cell suspension, and the cells were subjected to multiple *in vitro* and *in vivo* assays.

To evaluate the abundance of CSCs at the time of tumor removal, cells were stained for Aldefluor and analyzed by FACS. We found that VS-4718 treatment *in vivo* substantially decreased the percentage of Aldefluor+ cells in the MDA-MB-231 and SUM159 xenografts (Figure 2B, 2F) In contrast to VS-4718, treatment with paclitaxel did not change the proportion of Aldefluor+ cells (Figure 2B). Likewise, immunostaining of isolated tumors with an ALDH1A1-specific antibody demonstrated a reduction of ALDH1A1+ cells for the VS-4718 treated animals (Figure 2C, 2G). These experiments suggest that VS-4718 preferentially targets CSC *in vivo*.

To assess the self-renewal potential of cells after *in vivo* treatment with VS-4718, viable cells dissociated from tumors were plated in tumorsphere assays as above. Primary spheres were then dissociated into single cells and re-plated to gauge the ability to form secondary and tertiary tumorspheres. While the tumorsphere-forming efficiency in the controls remained similar in primary, secondary and tertiary tumorsphere assays, in both the MDA-MB-231 and SUM159 models, *in vivo* treatment with VS-4718 progressively decreased tumorsphere-forming efficiency to 100 spheres per 2000 cells for MDA-MB-231 tumor cells in a tertiary tumorsphere assay vs. 420 spheres in the vehicle control group (Figure 2D) and to 10 spheres per 1000 cells in a secondary tumorsphere assay for SUM159 cells vs. 130 spheres in the vehicle control group (Figure 2H). Paclitaxel did not alter the tertiary tumorsphere-forming efficiency of MDA-MB-231 tumor cells (Figure 2D). Therefore, *in vivo* treatment with VS-4718 preferentially reduced CSCs and self-renewal potential, while paclitaxel failed to do so.

The most rigorous and functional test for CSCs is the assessment of tumor-initiating capability following implantation of tumor cells in limiting dilutions into immunodeficient mice. To this end, cells from tumors in VS-4718-, VS-6063-, paclitaxel- or vehicle-treated mice were injected into SHrN (Harlan) immunodeficient mice in limiting dilutions, and animals were monitored for tumor development for 10-12 weeks. As shown in Figure 2E, 2I, VS-4718 diminished the calculated frequency of tumor-initiating cells (TICs) by 8-fold compared to control MDA-MB-231 tumors and by 4-fold for SUM159 tumors, respectively. In a separate independent experiment, the FAK VS-6063 inhibitor also decreased TICs by more than 8-fold compared to control tumors. In contrast, paclitaxel treatment increased the fraction of TICs 28-fold (Figure 2E). Collectively, these results indicate that treatment of tumor-bearing mice with the FAK inhibitors VS-4718 or VS-6063 clearly reduced the number of tumor-initiating cells *in vivo* in sharp contrast to chemotherapy which markedly increased the number of tumor-initiating cells in the tumors of treated mice.

### VS-4718 inhibits Wnt/β-catenin signaling

**Figure 3:**
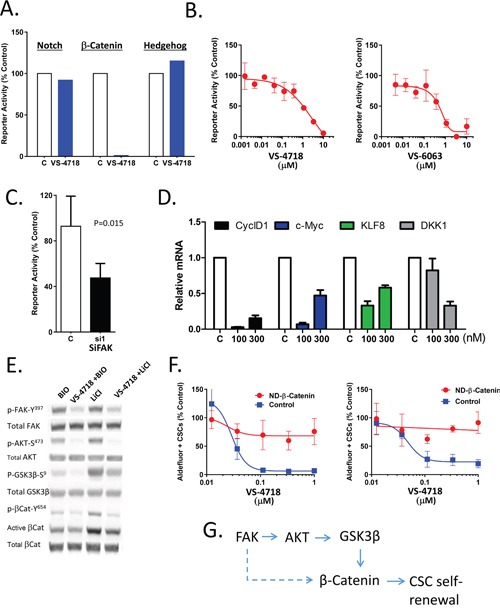
Inhibition of FAK attenuates Wnt/β-catenin signaling **(A-C)** FAK inhibition reduces reporter activity of Wnt/β-Catenin but not that of Notch or Sonic Hedgehog. **(A)** SUM159 cells were transfected with TOP/Flash β-Catenin/TCF, Notch (CSL) or Sonic Hedgehog reporter constructs and treated with VS-4718 (10μM) in matrigel for 24 h. Luciferase activity was measured by OneGlo assay (Promega). **(B)** MDA-MB-231 cells were infected with TOP/Flash reporter lentivirus and briefly selected with puromycin. Reporter cells were plated in matrigel and treated with VS-4718 or VS-6063 and stimulated with Wnt3A for 24h. OneGlo luciferase assay was performed. **(C)** Reporter cells, as in **(B)**, were transfected with siRNA against FAK or control, plated in matrigel and stimulated with Wnt3A for 24h. OneGlo luciferase assay was performed. **(D)** VS-4718 inhibits the expression of β-catenin/TCF target genes. SUM159 cells were treated with VS-4718 at indicated concentrations in matrigel for 2 days. Total RNA was subjected to RT-PCR for the indicated genes. **(E)** VS-4718 inhibits p-AKT, p-GSK3β and tyrosine phosphorylation of β-catenin. SUM159 cells were grown as tumorspheres for 3 days and then treated with VS-4718 and simultaneously stimulated with either Bio or LiCl for 4h. Cell lysates were analyzed by immunoblotting for indicated proteins. **(F)** Non-degradable β-catenin overcomes the CSC-targeting inhibitory effect of VS-4718. MCF7 and SUM159 cells were transfected with a control plasmid or a plasmid encoding T41A mutant form of β-catenin that is non-degradable. Stable cell lines were established after G418 antibiotic selection. Cells were treated with VS-4718 in matrigel for 4 days and Aldefluor assay was carried out. **(G)** Inhibition of FAK kinase activity targets CSC self-renewal through β-Catenin dependent mechanism. Data presented in this figure are representative of 2 or 3 independent experiments.

The aberrant activity of several developmental pathways has been implicated in maintenance of CSCs and cancer development. To probe into possible interplay between FAK and developmental signaling pathways, we employed reporter assays for Wnt, Notch and Hedgehog pathways so that the involvement of these pathways could be monitored in the context of FAK inhibition. We observed that VS-4718 treatment of transfected breast cancer lines induced nearly complete reduction in Wnt/β-catenin TOPFLASH reporter activity, while having no effect on either Notch or Hedgehog reporters (Figure 3A). Since the structurally distinct FAK inhibitors VS-4718 and VS-6063 both dose-dependently inhibited Wnt/β-catenin reporter activity, it suggests that FAK kinase activity is required for Wnt/β-catenin function (Figure 3B). Similarly, siRNA-mediated FAK knock down suppressed Wnt/β-catenin reporter activity by 50% (Figure 3C). Accordingly, VS-4718 treatment also reduced the expression levels of β-catenin target genes, CyclD1, c-Myc, Klf8, and DKK1, as measured by qRT-PCR (Figure 3D), further supporting the notion that inhibition of FAK activity attenuates activation of downstream Wnt/β-catenin signaling.

Tyrosine phosphorylation has been reported to modulate β-catenin activity. Specifically, phosphorylation of β-catenin at its tyrosine 654 residue has been shown to activate Wnt/β-catenin signaling and promote intestinal tumorigenesis [33]. FAK is also known to modulate the activity of both AKT and GSK-3β. FAK promotes the phosphorylation and activation of AKT, which in turn phosphorylates GSK-3β on the serine 9 residue and inactivates GSK-3β; the latter phosphorylates β-Catenin, driving its degradation [34]. In our own hands, we cultured MDA-MB-231 cells as tumorspheres for 3 days to enrich for CSCs and then treated with VS-4718. β-catenin signaling was induced by either LiCl or BIO compound (both selective GSK-3β inhibitors [35, 36] that act by sparing β-catenin from the phosphorylation that otherwise drives its degradation). Interestingly, VS-4718 treatment decreased S^473^ phosphorylation of AKT, S^9^ phosphorylation of GSK-3β and Y^654^ phosphorylation of β-catenin (Figure 3E). A corresponding decrease of active β-catenin was also observed while total β-catenin remained unchanged (Figure 3E). This suggested that FAK impinges on β-catenin activity either through direct Y^645^ phosphorylation of β-catenin or/and indirectly through regulation of AKT/GSK-3β signaling (Figure 3G).

We reasoned further that if the inhibitory effect of FAK inhibitors on CSCs is mediated through β-catenin, such an effect should be abolished by ectopic expression of a constitutively active mutant form of β-catenin. To test this hypothesis we employed a previously characterized in a subset of breast cancers [37, 38] non-degradable, constitutively active form of β-Cat (T41A). The effect of VS-4718 on Aldefluor+ CSC abundance was compared in MCF7 and SUM159 cells transfected with a non-degradable mutant β-catenin (T41A) and vector control. The expression of constitutively active T41A β-catenin abolished the inhibitory effect of VS-4718 on Aldefluor+ cells (Figure 3F), suggesting that FAK inhibition reduces CSC maintenance at least partially through downstream inhibition of Wnt/ß-catenin signaling.

### FAK inhibition extends the response to chemotherapy

**Figure 4:**
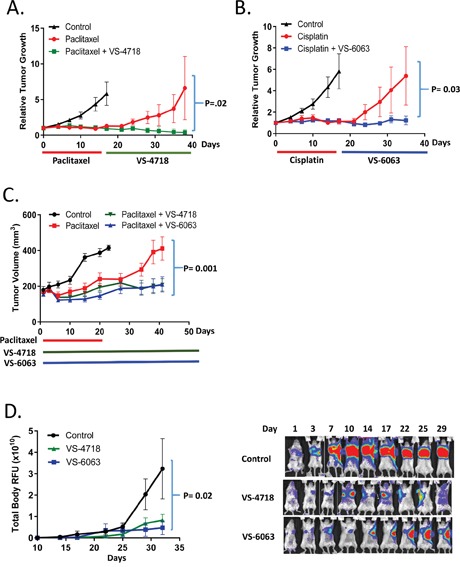
FAK inhibitors VS-4718 and VS-6063 delay tumor re-growth and development of metastasis **(A-C)** VS-4718 and VS-6063 delay tumor re-growth after paclitaxel or cisplatin treatment. **(A, B)** Mice bearing CAL-51 TNBC xenograft tumors were treated with paclitaxel (10 mg/kg, q3d 6x) or cisplatin (5 mg/kg, q3d 6x) followed by control, VS-4718 (50 mg/kg, bid, 20 days) or VS-6063 (50 mg/kg, bid, 20 days). Tumor volume was measured twice a week. **(C)** Mice bearing PDX model TNBC-607 (Molecular Response) were treated with paclitaxel (10 mg/kg, q7d, 3x), paclitaxel+VS-4718 (50 mg/kg, bid, 40 days) or paclitaxel+VS-6063 (50 mg/kg, bid, 40 days). Tumors were measured once a week. **(D)** VS-4718 and VS-6063 inhibit the outgrowth of metastasis in the 4T1-Luc TNBC model. Primary tumors were surgically removed at day 10 after inoculation; mice were then randomized into 3 groups (n=12) and treated with VS-4718 (50 mg/kg, bid, 20 days) or VS-6063 (50 mg/kg, bid, 20 days). Luciferin was injected twice weekly and mice were imaged using the IVIS imaging station (Perkin Elmer, at Biomodels, Watertown, MA). Representative images of 3 mice during the treatment are shown on the right. Images were taken with the same exposure parameters during the entire study.

An increased percentage of CSCs in patients with breast cancer following neoadjuvant chemotherapy has been observed [5]. Moreover, CSCs have been postulated to be responsible for tumor recurrence after initial rounds of chemotherapy [39]. We hypothesized that by targeting CSCs, FAK inhibitors may delay tumor regrowth following cessation of chemotherapy. To test this concept, mice bearing CAL-51 triple-negative breast cancer (TNBC) tumors were treated with cisplatin or paclitaxel to debulk tumors, and subsequently treated with VS-4718, VS-6063 or vehicle control. We chose doses of paclitaxel and cisplatin where tumor growth was initially inhibited, but the CAL-51 tumors regrew quickly after cessation of chemotherapy. Importantly, treatment with FAK inhibitors, VS-4718 or VS-6063, substantially delayed tumor regrowth in all treated mice (Figure 4A, 4B). Using a second animal model with a patient-derived xenograft (TNBC-607; Molecular Response), concomitant treatment of mice bearing human TNBC tumors with either paclitaxel and VS-4718 or paclitaxel and VS-6063 followed by single agent FAK inhibitor treatment showed that both FAK inhibitors significantly prolonged progression-free survival of these animals (Figure 4C). These results are consistent with the reduction of chemotherapy-resistant CSCs by FAK inhibitors, resulting in a longer delay of tumor regrowth following cessation of chemotherapy treatment.

Tumor initiation at a distant site is an important step in metastasis, which is a major cause of cancer mortality. FAK has been shown to be important for tumor initiation and the outgrowth from micrometastases to macrometastases [40]. Accordingly, we sought to determine if FAK kinase inhibitors can impede the outgrowth of metastases. To do so, we studied the 4T1 murine TNBC model, which spontaneously metastasizes to lymph nodes, lungs and other organs following orthotopic implantation in the mammary fat pad. Luciferase-labeled 4T1 cells were implanted in 5^th^ mammary fat pad of syngeneic Balb/c female mice. After reaching approximately 100 mm^3^ in size (10 days after implantation), primary tumors were surgically removed to model adjuvant treatment. In this manner the progression of tumor development to other sites was not dictated by continual seeding from the mammary fat pad. Following the mammary fat pad excision, mice were treated with vehicle, VS-4718 or VS-6063 each at 50 mg/kg bid, and the progression of metastasis was monitored by bioluminescence imaging (Figure 4D). 100% of mice in the control group developed metastases with progressive outgrowth post-surgery. Both FAK inhibitors VS-4718 or VS-6063 caused significant delay in metastatic growth indicated by significantly (p=0.02) reduced values of chemoluminescence (RFU) at the metastatic sites (Figure 4D). These results provide evidence that FAK inhibitors can delay metastatic outgrowth.

## DISCUSSION

Several studies with genetic ablation have implicated FAK in cancer initiation, progression and metastasis, and increased FAK activity and expression in tumors have been linked to poor prognosis [16]. Moreover, integrin/FAK signaling has been implicated in the maintenance of breast cancer stem cells [26]. Accordingly, FAK has been pursued as an attractive anti-cancer drug target [19, 41]. FAK kinase function is important for CSC in basal-like TNBCs and high grade DCIS, while a kinase-independent function may be important for maintenance of CSC in claudin-low breast cancers [27, 42], a subset of breast cancer accounting for about 7-14% of TNBC [43].

In the present study, we provide strong evidence that the pharmacological inhibition of FAK kinase by two structurally distinct small molecule kinase inhibitors, VS-4718 and VS-6063, leads to attenuation of CSC function, both *in vitro* and *in vivo*. Given the complexities surrounding CSC markers, our approach was to employ multiple orthogonal CSC assays to demonstrate the effect of FAK inhibition on CSCs. *In vitro*, *in vivo*, and *ex vivo* treatment of breast cancer cell lines and patient samples with either VS-4718 or VS-6063 led to preferential reduction of CSC subpopulations as demonstrated by 1) decrease of the proportion of Aldefluor+ and SP cells, 2) attenuation of tumorsphere-forming efficiency, and 3) reduction of tumor-initiating capacity as measured by re-implantation in limiting dilution assays. In contrast to CSC markers, which may vary in different cancer types or represent only subsets of tumor-initiating cells, limiting dilution of cells followed by measurement of *in vivo* tumor-initiating ability represents a reliable metric that gauges a fundamental trait of CSCs. In our work, both SUM159 and MCF7 CSCs were demonstrated to be inhibited by the FAK inhibitors. SUM159 and MCF7 cell lines, which are derived from different breast cancer subtypes, were both inhibited in CSC assays by VS-4718 and VS-6063. Our findings suggest that the multiple CSC populations emanating from the breast cancers of different origin are controlled by FAK, indicating its importance in a central pathway of CSC maintenance and survival.

Numerous studies have indicated that different forms of chemotherapy or radiation will differentially block proliferating tumor cells and spare CSCs. Recently, it was shown that high grade breast DCIS contains CSCs that are resistant to radiotherapy [42]. Indeed, recent clinical studies indicate that paclitaxel, a component of neoadjuvant chemotherapy for breast cancer, increases the percentage of CSCs in residual tumors [5]. Importantly, breast cancer patients receiving taxane-based chemotherapy had a worse prognosis if ALDH+ CSC-positive residual disease was detected, while patients with CSC-negative residual disease have a prognosis similar to that of patients with pathologic complete response [4, 5]. The increase in the fraction of CSCs following treatment with chemotherapy may occur passively, as a consequence of poor elimination of CSCs, or actively, through stimulation of cytokines which in turn stimulate CSC proliferation and self-renewal [44]. Cells surviving chemotherapy show gene expression profiles similar to that of cells undergoing EMT, a process known both to generate CSCs and to rely on FAK as an important pro-survival factor [40]. Our data indicate that VS-4718 or VS-6063 can counteract chemotherapy-induced enrichment of CSCs *in vitro*, by overcoming paclitaxel resistance in a breast cancer cell line (Figure 1G). The preferential induction of apoptosis in CSCs by VS-4718 (Figure 1H) suggests that CSCs may be more dependent on FAK signaling than non-CSCs, and argues for a CSC-intrinsic role of FAK.

Our studies in breast cancer cell lines also contribute to the previously proposed crosstalk between the FAK and Wnt/β-catenin pathways [37, 45]. Aberrant activation of the Wnt/β-catenin pathway leads to cancer development. Integrin-FAK activity leads to activation of PI3K/AKT signaling and, in turn, phosphorylation of the S9 residue of GSK-3β [34]. This phosphorylation inhibits the activity of GSK-3β which would otherwise drive rapid degradation of β-catenin. Thus, FAK appears to be a positive regulator of Wnt/β-Catenin. A connection between FAK and β-Catenin was suggested in keratinocyte stem cells [46]. FAK also regulates transcription of several components of the Wnt/β-Catenin pathway: Wnt3a [47], Frizzled, DKK1, and LRP5 [48]. Our results show that the inhibition of FAK kinase activity leads to inhibition of phosphorylation of β-catenin on Y^654^. Previous studies have revealed that Y^654^ is phosphorylated by activation of receptor-tyrosine kinases such as cMet and RON and this phosphorylation leads to both the release of β-catenin from cadherins and activation of TCF-transcriptional activity [51]. Phosphorylation of Y^654^ on β-catenin has also been linked to increased motility and invasion of cancer cell lines and the heterozygous phosphomimetic E^654^ mutant β-catenin is sufficient to promote spontaneous intestinal tumorigenicity [33]. In support of the hypothesis that FAK regulates the self-renewal of CSCs at least in part through activating β-catenin, expression of a constitutively active mutant β-catenin abolished the reduction of CSCs by FAK inhibition. Our data that FAK kinase inhibitors decreased both phosphorylation on Y^654^ and active β-catenin suggests a mechanistic explanation for why FAK is required for the maintenance of CSCs.

In a variety of preclinical models, FAK mediates resistance to paclitaxel, pemetrexed, dasatinib, and BRAF inhibitors [21, 23, 29, 49–51]. Since there are numerous examples of FAK pathways driving the invasiveness of cancer, undoubtedly the roles of FAK extend to additional attributes of the tumor microenvironment beyond cell-intrinsic CSC properties. Addition of FAK inhibitors to chemotherapy *in vivo* has the net benefit of reduced tumor growth and more sustained response that far exceeds the effect of chemotherapy alone (Figure 4A–4C). Since the development of metastasis is also inhibited by VS-4718 or VS-6063 as single agents (Figure 4D), there is a compelling rationale for involvement of FAK inhibitors impacting multiple stages of tumor progression. In any case, the combination of FAK inhibitors with chemotherapy, enabling effective targeting of both CSCs and non-CSCs, could prevent or substantially delay primary tumor growth and metastatic outgrowth.

Our findings have significant implications for the clinical development of FAK inhibitors. Current first-line chemotherapy generally consists of cytotoxic agents, such as taxanes, platinum agents (e.g. cisplatin), and antimetabolites. While these agents may be effective at debulking tumors and controlling disease initially, disease relapses commonly occur. Since these cytotoxic agents increase the proportion of CSCs, a CSC-targeting agent is expected to block CSCs from regenerating the tumor burden, thereby extending the duration of response and preventing tumor recurrence. Elimination of CSCs remaining after chemotherapy may impede cancer recurrence and lead to sustained antitumor response for patients. Numerous clinical trials across various cancer types with anti-CSC agents have been launched [13]. Many of these novel agents may be expected to be most effective in combinations with standard chemotherapy, as we have demonstrated here with FAK inhibitors. Our findings provide strong rationale for the clinical development of FAK inhibitors for the treatment of cancer with the goal of achieving more durable responses through the preferential targeting of cancer stem cells.

## MATERIALS AND METHODS

### Cell lines, primary human tumor tissues, compounds and reagents

MDA-MB-231 and MCF7 cell lines were obtained from ATCC and were cultured using ATCC recommended media. SUM159 cells were acquired from Asterand (Detroit, MI) and maintained in F12 medium supplemented with 10% fetal bovine serum (FBS), 5 μg/ml insulin, 1 μg/ml hydrocortisone, and penicillin (100 units/ml)/streptomycin (100 μg/ml). CAL-51 cells were obtained from DSMZ (Braunschweig, Germany) and grown in DMEM medium supplemented with 20% FBS, and penicillin (100 units/ml) /streptomycin (100 μg/ml). 4T1-Luc2 cells were obtained from Perkin Elmer (Waltham, MA). Cell lines used were authenticated by STR (short tandem repeat) analysis at ATCC with the exception of SUM159 and CAL-51 for which STR analysis was done at IDEXX Radil (Columbia, MO). Primary human breast tumor specimens were obtained from Tissue Solutions Ltd. (Glasgow, UK) after patient's consent and institutional IRB approval. Tumor fragments were placed in tumorsphere-forming medium [31] and treated with compounds for 4 days and subjected to CSC assays. Anti-CD44 and -CD24 antibodies were purchased from BD Biosciences (San Jose, CA). Anti-EpCAM, biotin- anti-mouse CD45, and biotin-anti-mouse H-2K were purchased from BioLegend (San Diego, CA), anti-p-Y654β-catenin was purchased from ECM Bioscience (Versailles, KY), and antibodies to active- and total-β-catenin and β-actin were purchased from Cell Signaling Technologies (city, MA). VS-4718 was synthesized by Poniard. VS-6063 was synthesized by Paraza Pharma (Montreal, QC). Paclitaxel, cisplatin and carboplatin were purchased from Selleckchem (Munich, Germany). LiCl was purchased from Sigma-Aldrich (St. Louis, MO) and BIO was purchased from Tocris (Bristol, UK). Luciferase reporter constructs (Wnt/β-Catenin TOPFLASH, Notch, and Hedgehog) were purchased from Qiagen and transfected using Superfect reagent (Qiagen). Mutant β-Catenin construct (RC400227) was purchased from OriGen (Rockbille, MD). Stable cell lines expressing β-Catenin T41A were generated after MCF7 and SUM159 cells were transfected with mutant β-Catenin construct or vector control and selected with 0.8 mg/ml G418 (Sigma). siRNA against FAK, PCR primers for FAK, Myc, CyclD1, c-Myc, KLF8 were purchased from Qiagen. Total RNA was prepared using RNeasy kit (Qiagen). cDNA was synthesized from 1 μg total RNA using First Strand kit (Qiagen) and RT-PCR was performed using SYBR Green reagent (Qiagen).

### Cell dissociation from tumor tissue

Human tumor fragments were received under approved protocols from the vendor (Tissue Solutions LTD, Manchester UK). To dissociate samples into single cells, xenograft tumors or human tissue fragments were minced into smaller pieces and incubated in HEPES-Eagle's medium containing either 50 μg/ml Liberase (Roche Applied Science, Indianapolis, IN) for xenograft models or a mixture of 50 μg/ml collagenase and 50 μg/ml dispase for human tumors for 1h at 37°C under agitation. Tissue fragments were triturated by pipetting through pipettes with progressively smaller orifices (25, 10 and 5 ml) every 15 minutes. Dissociated cells were filtered through a 100 μm nylon mesh, centrifuged at 800 x g for 5 min and washed 3 times in RPMI medium supplemented with 20% FBS, 100 U/ml penicillin, and 100 μg/ml streptomycin. To remove mouse cells from tumor xenografts, 2×10^6^ live cells were suspended in 1 ml RPMI supplemented with 10% FBS. Ten μl of biotin anti-mouse CD45 and 10 μl biotin anti-mouse H-2Kd were added and incubated for 15 min on ice with occasional rocking. Fifty μl washed Dynabeads Biotin Binder (Invitrogen) were added and incubated for 20 min on ice with occasional rocking. Beads were separated with magnet and free cell suspension transferred into new tubes.

### Matrigel on top (MoT) culture

Matrigel-on-Top (MoT) assay was previously described [29].

### Aldefluor assays

An imaging-based Aldefluor assay was carried out using the Aldefluor assay kit (STEMCELL Technologies, Vancouver, BC) as described in [29]. Briefly, 30 μl of the aldefluor buffer containing aldefluor reagent and 10 ng/ml Hoechst 3342 was added in each well and incubated at 37°C for 20min. Wells were washed with aldefluor buffer and plates were imaged using Celigo (Brooks Life Science Systems, Chelmsford, MA) and Aldefluor-positive and total cells were quantitated. Each plate contained wells treated with ALDH inhibitor diethylamino benzaldehyde (DEAB), used as a negative control. Those wells were served to set gate (<0.05% ALDH+ cells) in the quantitative analysis.

For FACS-based Aldelfuor assay, cells dissociated from tumors were stained with the Aldefluor kit according to manufacturer's recommendations (Stemcell Technologies). Viable cells were determined after 7-AAD staining (BD Biosciences). The percentage of live Aldefluor+ cells was quantified by FACSCalibur (BD, Franklin Lakes, NJ). A portion of cells were incubated with the ALDH inhibitor, diethylamino benzaldehyde (DEAB), to serve as a negative control for setting gate for FACS analysis.

### Side population (SP) analysis

Hoechst 33342 exclusion (Side Population) assay was carried out as previously described [52].

### Tumorsphere assay

To determine tumorsphere forming efficiency, single cell suspension was plated in tumorsphere forming medium as previously described [31]. Spheres were enumerated using Celigo (Brooks Life Science Systems). For secondary and tertiary sphere formation, tumorspheres were dissociated by trypsin digestion and 2000/ml live cells were re-plated for secondary and tertiary tumorsphere assays.

### Apoptosis assays

Induction of apoptosis in Aldefluor+ and Aldefluor- cells was measured by Annexin V binding. Briefly, SUM159 cells were treated with VS-4718, paclitaxel or DMSO control for 24h. Cells were first stained with Aldefluor reagents for 30 min followed by Annexin V-PE (EBioscience, San Diego, CA) and propidium iodide staining for 10 min. Percentage of pro-apoptotic AnnexinV+ cells was determined in proportion to PI-negative (live) cells.

### Tumor xenograft, tumor-initiating, and metastasis studies

Xenograft and *in vivo* tumor initiating studies were conducted at Avastus Preclinical Services (Cambridge, MA) and in compliance with Institutional Animal Care and Use Committee (IACUC) protocol. *In vivo metastasis* model and bioluminescent studies were conducted at Biomodels LLC. (Watertown, MA) and in compliance with IACUC protocol. Briefly, 10^6^ 4T1-Luc2 cells were implanted in the 5^th^ mammary fat pad of Balb/c mice. Primary tumors were excised when they reached 100-150 mm^3^ average size.

*Xenografts*: 4×10^6^ MDA-MB-231 or SUM159 cells admixed with matrigel (BD Biosciences) were injected orthotopically in the mammary fat pad of 6-8-week-old female Nu/Nu mice. After tumors reached an average size of 100-150 mm^3^ at approximately 3-6 weeks, mice were randomized into groups and treated with either vehicle alone, 100 mg/kg VS-4718 bid by oral administration for 10 days, paclitaxel 10 mg/kg or carboplatin 15 mg/kg ip. Tumors were harvested 2h post last dose and kept in ice-cold PBS.

*Tumor initiating assessment*: Cells dissociated from MDA-MB-231 or SUM159 tumors were admixed with matrigel and injected into the mammary fat pad of female immunodeficient SHrN mice (Harlan Laboratories). Tumor formation was assessed 8-12 weeks after implantation. Tumor-initiating cells (TIC) was calculated using ELDA software (http://bioinf.wehi.edu.au/software/elda/).

*In vivo* *metastasis* model and bioluminescent studies were conducted at Biomodels LLC. (Watertown, MA). Briefly, 10^6^ 4T1-Luc2 cells were implanted in the 5^th^ mammary fat pad of Balb/c mice. Primary tumors were excised when they reached 100-150 mm^3^ average size. Metastasis growth was monitored by luminescence imaging using the IVIS imaging station (Perkin Elmer, Waltham, MA) according to manufacturer's instructions.

## Abbreviations

CSC: cancer stem cell
FAK: focal adhesion kinase
TNBC: triple negative breast cancer
ALDH: aldehyde dehydrogenase
SP: side population
TICs: tumor initiating cells
PDX: patient-derived xenograft
DCIS: ductal carcinoma *in situ*.
